# 肺上皮干/祖细胞种类和研究方法进展

**DOI:** 10.3779/j.issn.1009-3419.2017.02.08

**Published:** 2017-02-20

**Authors:** 敏华 邓, 进华 李, 烨 甘, 平 陈

**Affiliations:** 1 100088 北京，中国人民解放军火箭军总医院呼吸内科 Department of Respiratory Medicine, PLA Rocket Force General Hospital, Beijing 100088, China; 2 410011 长沙，中南大学湘雅二医院呼吸内科 Department of Respiratory Medicine, Second Xiangya Hospital, Central South University, Changsha 410011, China; 3 410011 长沙，中南大学湘雅二医院康复科 Department of Rehabilitation, Second Xiangya Hospital, Central South University, Changsha 410011, China

**Keywords:** 肺上皮干细胞, 肺上皮祖细胞, 表型, 肺癌干细胞, Lung epithelial stem cells, Lung epithelial progenitor cells, Phenotype, Lung cancer stem cells

## Abstract

分离和鉴定肺上皮干/祖细胞，深入了解他们在肺脏生理病理条件下的具体作用机理，对于防治包括肺癌在内的肺脏疾病有重要意义。本综述介绍了已鉴定的肺上皮干/祖细胞种类和肺上皮干/祖细胞研究方法的最新进展，前者具有区域特异性，主要包括位近端气道的基底细胞和导管细胞，位细支气管的Clara细胞、变异Clara细胞、细支气管肺泡干细胞和诱导出的krt5^+^细胞及位肺泡的Ⅱ型肺泡上皮细胞和Ⅱ型肺泡上皮祖细胞；后者主要包括肺损伤模型、谱系示踪技术、三维培养技术、移植、慢性标记细胞法及单细胞转录组学分析等。最后简述了肺上皮干/祖细胞与肺癌的关系以及肺癌干细胞靶向药物治疗进展。

## Abstract (en)

Isolation and characterization of lung epithelial stem and progenitor cells and understanding of their specific role in lung physiopathology are critical for preventing and controlling lung diseases including lung cancer. In this review, we summarized recent advances in classification and research methods of lung epithelial stem and progenitor cells. Lung epithelial stem and progenitor cells were region-specific, which primarily included basal cells and duct cells in proximal airway, Clara cells, variant Clara cells, bronchioalveolar stem cells and induced krt5^+^ cells in bronchioles, type Ⅱ alveolar cells and type Ⅱ alveolar progenitor cells in alveoli. The research methods of lung epithelial stem and progenitor cells were mainly focused on lung injury models, lineage-tracing experiments, three dimensional culture, transplantation, chronic labeled cells and single-cell transcriptome analysis. Lastly, the potential relationship between lung epithelial stem and progenitor cells and lung cancer as well as lung cancer stem cell-targeted drug development were briefly reviewed.

因为肺与外界相通，所以肺上皮细胞持续暴露在过敏原、污染物、刺激物、烟雾、细菌/病毒感染等损伤因子下。若肺上皮细胞损伤和再生修复能力失衡，则导致疾病的发生发展。气道上皮在刺激损伤后的修复过程可分为3个相辅相成的步骤：①邻近上皮细胞通过局部扩散和迁移覆盖受损裸露区域；②肺上皮干/祖细胞通过迁移和增殖重建上皮；③上皮干/祖细胞分化为各种特定细胞以修复气道屏障和呼吸功能^[1]^。肺上皮干/祖细胞在维持肺内环境的稳态和损伤后的再生修复中起重要作用，分离和鉴定肺上皮干/祖细胞，深入了解他们在肺脏生理病理条件下的具体作用机理，对肺脏疾病的防治意义深远。肺干细胞是存在于肺组织内的成体干细胞，他们在稳态下以极低的频率分裂，具有长期自我更新和产生一种或多种高度分化功能细胞的能力；肺祖细胞也能分化为一种或多种高度分化细胞，但是通常被认为仅具有有限的自我更新能力。由于目前尚无公认的可评估自我更新能力的体内或体外实验方法来区别肺干细胞和祖细胞，并且这种自我更新能力可能受微环境因素的影响，所以学者们多将肺干细胞和祖细胞一并讨论。肺内的干/祖细胞包括肺上皮干/祖细胞、肺间充质干细胞和肺内皮祖细胞等，而且多种肺脏难治性疾病如肺癌、慢性阻塞性肺疾病、哮喘、肺囊性纤维化、特发性肺纤维化等都可能与肺干/祖细胞的调节和功能异常密切相关。本文就已经鉴定的肺上皮干/祖细胞的种类、肺上皮干/祖细胞的研究方法以及他们和肺癌的关系的最新进展进行综述。

## 1 肺上皮干/祖细胞种类

肺上皮干/祖细胞可能是兼职（faculative）干/祖细胞，即在正常情况下处于静息状态并参与肺脏呼吸功能，如一些干/祖细胞分泌对气体交换至关重要的蛋白，但是当肺损伤时则迅速变为短暂扩充细胞参与损伤后的再生修复^[2]^。由不同类型的细胞、多种细胞外基质蛋白和一些生长因子组成的微环境构成“干细胞龛位（stem cell niches）”，这种龛位对于决定干/祖细胞的功能和分化效能起关键作用^[3]^。大多数学者认为肺内存在多种位于特定区域龛位内的上皮干/祖细胞，不同的肺上皮干/祖细胞在稳态和损伤修复反应中可能发挥各自特定的作用，不同类型或程度的损伤启动不同的肺上皮干/祖细胞参与修复^[4]^。表 1列出了主要的肺上皮干/祖细胞及其表面标记，我们将按这些干细胞龛位所在部位的顺序对肺上皮干/祖细胞进行阐述。

**1:** 小鼠主要的肺上皮干/祖细胞 Major lung epithelial stem and progenitor cell types in adult murine

Cell type	Cellular marker
Basal cells	Trp63^+^, Krt5^+^, Krt8^+^, Krt14^+^, NGFR^+^, AQP3^+^
Duct cells	Krt5^+^, Krt14^+^
Clara cells	CCSP^+^
vClara cells	EpCAM^+^/Sca-1^lo^/AF^lo^/CCSP^+^
BASCs	CCSP^+^/SPC^+^, CD24^lo^/Sca-1^lo^/EpCAM^+^/integrin*α*6^+^
PNECs	CGRP^+^
Induced krt5^+^ cells or DASCs or LNEPs	Trp63^+^/Krt5^+^
ATII cells	Pro-SPC^+^
ATII progenitor cells	Integrin *α*6^+^*β*4^+^
Trp: Transcription factor；Krt: Keratin; NGFR: Nerve growth factor receptor; AQP: Aquaporin; AF: Auto-Fluorescence; lo: Low expression; CCSP: Clara cell secretory peptide, also called Scgb1a1 (secretoglobin family 1A member) or CC10; Sca-1: Stem cell antigen-1; CGRP: Calcitonin gene related protein; SPC: Surfactant protein C; vClara cells: variant Clara cells; BASCs: Bronchioalveolar stem cells; PNECs: Pulmonary neuroendocrine cells; DASCs: Distal airway stem cells; LNEPs: Lineage-negative epithelial progenitor cells; ATII cells: Type Ⅱ alveolar cells.	

### 1.1 近端气道（气管-支气管）肺上皮干/祖细胞

基底细胞（basal cells, BCs）是在小鼠的气管和近端气道以及人类的全气道的稳态与损伤后再生修复中起重要作用的干/祖细胞。将经过荧光激活细胞分选技术（fluoresence-activated cell sorting, FACS）分离的BCs（Trp63^+^NGFR^+^Krt5^+^）在基质胶中培养可形成克隆结构，并且这种克隆结构表达纤毛细胞和Clara细胞的表面标记。谱系示踪技术也证明，BCs可自我更新并在稳态与SO_2_吸入损伤后分化为纤毛细胞和Clara细胞^[5]^。最近，Watson等^[6]^利用长期谱系示踪技术和单细胞基因转录组学分析发现基底细胞是由数量相近的基底干细胞（basal stem cells, BSCs）和基底腔祖细胞（basal luminal precursor cells, BLPCs）组成。他们都表达Trp63和Krt5，几乎不表达Krt14，但是在受损后Krt14却迅速表达上调，这提示Krt14可能是在再生上皮中鉴定活化干细胞的一个重要分子标记。在稳态环境下，BSCs通过不对称分裂生成一个新的BSC和一个BLPC，后者可进一步分化为Clara细胞或神经内分泌细胞。BLPCs的自我更新速率很低甚至可忽略，而且因为表达Krt8，有所区别于BSCs。有意思的是，Pardo-Saganta等^[7]^利用SO_2_损伤模型观察到在Krt8^+^祖细胞形成之前，Trp63^+^基底细胞群便分离出两个亚群，一个将分化为成熟Clara细胞，另一个将分化为纤毛细胞，而且这种现象并没有在稳态上皮中被观察到，这说明BCs在损伤后产生不同于稳态环境下的祖细胞亚群。基底细胞可以作为气道上皮的组织特异性干细胞，但是他们的异质性还有待于进行进一步研究^[8]^。

导管细胞是位于气管粘膜下腺体（submucosal glands, SMGs）导管部的多能干/祖细胞。Borthwick等^[9]^将导管细胞种植到去除上皮的气管基底膜上，再将其一起移植到免疫缺陷小鼠皮下，发现这些细胞可在裸露的气管内再生上皮样表面。在严重的缺血缺氧损伤后，导管细胞可分化为粘膜下腺小管、导管细胞和气道上皮细胞^[10]^。

### 1.2 细支气管肺上皮干/祖细胞

Clara细胞已被谱系示踪技术证明为可自我更新并能产生纤毛细胞的干/祖细胞^[11]^。在小鼠细支气管区的神经内分泌小体（neuroendocrine body, NEB）周围和细支气管肺泡连接处（bronchioalveolar duct junction, BADJ）分别代表两个不同的干细胞龛位。谱系示踪实验证明，CGRP^+^肺神经内分泌细胞（pulmonary neuroendocrine cells, PNECs）不仅在稳态下可以自我更新，而且在萘损伤后可分化为Clara细胞和纤毛细胞，但是PNECs的去除对Clara细胞修复并无明显影响^[12]^，这表明PNECs可能不是参与细支气管修复的主要细胞。NEB周围和BADJ都有变异Clara（variant Clara, vClara）细胞分布，他们是在萘损伤后位于终末细支气管最早而且最主要的增殖细胞，并且可以自我更新并能分化为Clara细胞^[13]^。Teisanu等^[14]^通过包括三维培养技术、谱系示踪技术等在内的多种技术，发现一种对萘耐受的细支气管祖细胞，他们是CD45^-^CD31^-^CD34^-^EpCAM^+^Sca-1^lo^AF^lo^细胞，而在CD45^-^CD31^-^CD34^-^EpCAM^+^Sca-1^lo^AF^hi^（hi：high，高表达）细胞群中包含对萘敏感的Clara细胞。BADJ区内还分布极少量对萘耐受的细支气管肺泡干细胞（bronchioalveolar stem cells, BASCs），他们共表达CCSP和pro-SPC，可在体外自我更新并能分化为Clara细胞、Ⅰ型肺泡细胞（type Ⅰ alveolar cell, ATI）和Ⅱ型肺泡细胞（type Ⅱ alveolar cell, ATII），但是不能分化为纤毛细胞^[15, 16]^。利用CD31^-^CD45^-^Sca-1^+^表型可将BASCs阳性富集^[17]^。然而，另一项研究结果^[15]^显示利用CD45^-^CD31^-^EpCAM^+^Sca-1^lo^CD24^lo^表型可富集BASCs。此外，McQualter等^[18]^采用FACS分选出肺CD45^-^CD31^-^EpCAM^hi^ CD49f^+^CD104^+^CD24^lo^细胞，将这些细胞进行三维培养时形成了表达气道和/或肺泡系标记的克隆。

Kumar等^[19]^报道了当亚致死剂量的H1N1流感病毒感染小鼠后，一群Trp63^+^Krt5^+^细胞迁移至细支气管及其周围肺泡区域，增殖修复受损的肺泡上皮。近期两篇发表于*Nature*的研究对此细胞的来源和作用机制进行了进一步探讨。虽然这些细胞，有些学者称之为远端气道干细胞（distal airway stem cells, DASCs）和气管基底干细胞具有类似的分子标记，但是却与基底干细胞在体外培养和体内移植实验中表现出不同特性。气管基底干细胞在体外和体内研究中生成的都是更接近近端的气道上皮，而这些细胞在体外可形成肺泡球状物，在体内可产生肺泡细胞和Clara细胞，并且krt5谱系研究表明这些细胞是在肺损伤后才产生的^[20]^。Zuo等^[20]^为了更进一步明确DASCs在H1N1所致肺损伤修复中的作用，利用转基因技术去除了小鼠的DASCs，发现相比于对照组小鼠，这些小鼠在感染H1N1后出现了肺功能下降并且富集的白细胞也未消散的情况。令人惊讶的是，将从健康小鼠分离出的DASCs经扩增后通过气管内滴注移植入小鼠肺内，上述现象得到了缓解。Zuo和Vaughan两者的团队^[20, 21]^都通过谱系示踪技术对于H1N1感染诱导出的krt5^+^细胞的细胞来源进行研究，但两者的结果却不一致。Zuo等^[20]^观察到超过50%的这些细胞来自于已存在的krt5^+^谱系细胞，而Vaughan的团队却只追踪到13%来源于krt5^+^谱系。这原因可能是两者采用不同的报告系统（Zuo等采用使用一个带有牛krt5启动子的转基因，而Vaughan等^[21]^是在内源性krt5基因的3’端未转译区敲入一个转基因）。Vaughan等的研究结果显示这些诱导出的krt5^+^细胞也不是来源于SPC^+^谱系或CC10^+^谱系，因而他们称之为谱系阴性上皮祖细胞（lineagenegative epithelial progenitor, LNEPs）。在Vaughan等的实验中这些细胞也出现于博来霉素损伤后，尽管数量程度低于流感病毒感染后，但是也提示krt5^+^再生过程并不是流感病毒感染后的特异性过程。Vaughan等还发现这些细胞可能来源于整合素β4^+^CD200^+^上皮干/祖细胞，这与Quantius等^[22]^的研究结果一致。他们观察到高致病性的流感病毒虽然可以感染小鼠肺内多种细胞群，但对一个具有高增殖能力的上皮细胞亚群（EpCAM^hi^CD24^low^integrinα 6^high^）表现出强烈的趋向性。这些细胞表达Sca-1，高度富集整合素β4^+^CD200^+^上皮干/祖细胞，且促进流感病毒感染后的Trp63^+^Krt5^+^再生过程。

### 1.3 肺泡上皮干/祖细胞

ATII细胞早于1974年就被鉴定为肺泡上皮的干/祖细胞^[23]^。Desai等^[24]^综合运用分子标记、谱系追示和三维培养等方法发现在胚胎肺发育过程中ATI和ATII细胞都来源于一种双能祖细胞，而在出生后新生的ATI细胞来自于少量的具有自我更新能力的成熟ATII细胞，并且ATII细胞的这种干细胞特性在ATI细胞受损后迅速被广泛地激活。Liu等^[25]^采用谱系示踪技术发现参与绿脓杆菌介导的肺泡损伤后修复的是ATII细胞的一种亚群，即Sca-1^+^ ATII细胞。这些细胞不表达整合素β4、Trp63和CCSP，是不同于其他远端气道祖细胞及Sca-1^+^ BASCs的。除了ATII细胞，还有其他干/祖细胞参与肺泡上皮的稳态维持和损伤后修复。利用谱系示踪技术在CCSP-CreER（Cre recombinase-modified estrogen receptor fusion protein）小鼠（CCSP驱动的可诱导性Cre重组酶，利用此基因重组小鼠可以谱系示踪CCSP^+^细胞）中，Rawlins等^[11]^发现在稳态或高氧损伤后的肺泡内，CCSP^+^细胞并没有增加，提示这种细胞不分化为肺泡上皮细胞；然而Rock等^[26]^报道当此种小鼠被博来霉素肺损伤后，CCSP^+^细胞分化为ATI和ATII两种细胞，提示CCSP^+^细胞如BASCs或Clara细胞，参与肺泡萘损伤后的修复。这两种不同的结论支持了前述观点，即不同类型或程度的损伤启动不同的肺上皮干/祖细胞参与修复。Chapman等^[27]^的研究表明在博来霉素肺损伤后再生的ATII细胞大部分是来源于肺泡上皮细胞的一种新亚群，这种新的祖细胞表达层粘连蛋白受体4（α6^+^β4^+^），但pro-SPC表达量很少或缺失，在体外克隆实验中可分化为pro-SPC^+^细胞，并在移植实验中能形成气道和肺泡结构。这种α6^+^β4^+^细胞可能与McQualter等^[18]^发现的CD45^-^CD31^-^EpCAM^hi^CD49f^+^CD104^+^CD24^lo^细胞相交叉。

以上实验表明，许多新发现的推定的肺上皮干/祖细胞具有异质性或处于争论状态，尚需多种体内和体外实验方法来进一步鉴定和验证，也提示我们不仅需要在表型上深入了解特异的细胞表面标记以利于分选和鉴定肺上皮干/祖细胞，还需要完善包括谱系示踪在内的功能性实验来评估细胞的增殖分化能力。

### 1.4 人肺上皮干/祖细胞

鼠类的肺脏与人类之间有相同的形态和功能，但也存在一些差异，如基底细胞仅分布于小鼠气管和主支气管上皮，而在人体可分布至细支气管；呼吸性细支气管在小鼠中缺如。目前关于人肺上皮干/祖细胞所知甚少。Fujino等^[28]^获得了人肺泡上皮祖细胞（alveolar epithelial progenitor cells, AEPCs），这些细胞拥有ATII细胞和间充质干细胞的一些基因，提示他们在肺泡上皮细胞和间充质干细胞之间的表型重叠。实事上，AEPCs能够在间质和上皮之间表型转换，说明他们在肺组织修复中的潜在能力。多参数流式细胞分析显示那些推测的人气道上皮干/祖细胞具有一些与鼠类对应干/祖细胞相似的表达谱，如人NGFR^+^α6^+^Trp63^+^基底细胞可分化形成由Krt14^+^Trp63^+^基底细胞、Krt8^+^管腔细胞和纤毛细胞组成的囊性类器官结构^[29]^。Kajstura等^[30]^报道c-kit^+^细胞在组织培养时可分化为内胚层和中胚层两种细胞系，并且人c-kit^+^细胞在注入经低温冷冻后的小鼠肺内后参与气道、肺泡和肺血管的修复。此研究结果还有待在稳态和损伤的情况下利用转基因小鼠和谱系示踪等多种方法行进一步验证。若以上得到证实，则推翻了目前被普遍接受的观点，即在肺内不存在一种单一的可分化形成平滑肌、血管、气道和肺泡的肺多能干/祖细胞。Oeztuerk-Winder等^[31]^也可能分离出一种新的人肺上皮干细胞即E-Cad/Lgr6^+^细胞，这种细胞在体内外实验中具自我更新和分化特性，并且将单个E-Cad/Lgr6^+^细胞移植入肾囊后形成分化的支气管肺泡组织并保留自我更新的能力。

## 2 肺上皮干/祖细胞的研究方法

由于肺脏复杂的内在结构以及尚未发现肺上皮干细胞特异的表面标记等原因，肺上皮干/祖细胞的分选和鉴定相比其他组织干细胞更具挑战性。目前肺上皮干/祖细胞的研究方法主要包括肺损伤模型、谱系示踪技术、三维培养体系、移植、单细胞转录组学分析等，这些方法在分离和鉴定肺上皮干/祖细胞的应用中并不是孤立存在，而是相互结合，相互验证的。

### 2.1 肺损伤模型

因为肺上皮更新缓慢，绝大多数肺上皮干/祖细胞处于静息状态G_0_期，所以通过建立不同的肺损伤模型来激活肺上皮干/祖细胞。每种模型引发特定的损伤修复过程从而显露出存活细胞的增殖分化潜能，同时也有助于了解肺上皮干/祖细胞在各损伤模型所代表的肺疾病的发生发展过程中的作用。萘可特异性杀伤大多数Clara细胞，继而对萘耐受的vClara细胞通过增殖、分化和自我更新恢复这些Clara细胞群。vClara细胞对萘耐受的原因是他们不表达可将萘代谢为毒性产物的细胞色素P450 Cyp2f2^[32]^。然而，关于在萘损伤后远端肺内vClara细胞是否是唯一能自我更新并参与修复的细胞群的问题尚待解决，于是Reynolds等^[33]^建立了肺内Clara细胞表达单纯疱疹病毒胸苷激酶的转基因CCtK小鼠品系，通过给予更昔洛韦可清除所有Clara细胞而不受Cyp2f2表达的影响，研究显示在所有Clara细胞被去除后，气道上皮不能修复再生。Hong等^[4]^利用CCtK小鼠结合后述的[^3^H]胸腺嘧啶标记法发现PNECs也能自我更新，但尚不足以修复气道上皮。这些结果提示vClara细胞是气道上皮干/祖细胞或对干/祖细胞的维持至关重要。博来霉素可致小鼠ATII和ATI细胞损伤，用于研究肺泡上皮细胞的干/祖细胞^[16, 27]^。Ding等^[34]^采用单侧肺切除术首次证明了上皮细胞和内皮细胞之间的相互作用启动和维持肺损伤后的肺泡再生。亚致死剂量的H1N1流感病毒感染小鼠后引起广泛的细支气管和肺泡损伤，导致Clara细胞、纤毛细胞和ATII细胞的损失，在随后的肺再生过程中发现Trp63^+^Krt5^+^细胞可能是远端气道干细胞^[19]^。

### 2.2 谱系示踪

谱系示踪是基于特定类型细胞和他的任一子代细胞持续表达可通过组织学或活体影像学方法检测的报告蛋白的一种技术^[35]^。此方法有双重目的：①在不同条件下（稳态或损伤）追踪被标记细胞及其子代细胞的命运，这就可能明确特定细胞或细胞群在相应条件下的发育潜能和命运；②检测随着时间推移，在这些标记的细胞群中有多少比例的细胞被维持或被稀释，由此可判断一个细胞群随着时间推移是自我维持还是更新于另外一种未被标记的细胞群。谱系示踪方法是目前追踪一种细胞及其子代细胞的最佳手段，可以评判一种细胞的功能到底是干细胞、短暂扩充细胞、祖细胞、自我更新的未分化细胞还是终末分化细胞。Rawlins等^[11]^在运用此方法追踪推定的可分化为Clara细胞和肺泡上皮细胞的BASCs的子代细胞时，发现这些细胞无论是在肺损伤后还是在正常胚胎肺发育过程中都只产生传导气道上皮而无肺泡上皮。这在很大程度上削弱了可分化为所有肺细胞类型的“单一肺多能干细胞”的概念。

### 2.3 三维培养体系

三维培养体系指将不同类型的细胞、不同三维结构的载体如基质胶和生长因子同时在体外培养，使细胞能在类似于机体内环境的三维空间结构中迁移、分化和生长，形成三维复合物。此方法已成为鉴定肺干/祖细胞的增殖、分化和自我更新特性及研究上皮细胞和基质间的细胞和分子交互作用机制等的一种重要工具^[36]^。深入了解干/祖细胞龛位和关键信号通路调节因子的作用有助于探索出最优载体，从而使此体系不断完善。将永生的人支气管上皮细胞^[37]^或新鲜分离的人近端气道上皮细胞^[38]^在基质胶中行三维培养时都可形成由分化的气道上皮细胞组成的三维复合类器官结构。目前应用较多的是球体培养体系（spheroid culture system）。Chimenti等^[39]^报道这种球体培养体系可以形成对人肺祖细胞具有类似于龛位功能的微环境，能够影响成熟肺祖细胞的表型，并且还能分泌特定的旁分泌因子。

### 2.4 移植

肾囊移植模型用于在体内检测干细胞自主特性^[30]^。评估祖细胞潜能的新方法还有用基质胶皮下注射肺多能干细胞^[40]^和体外去细胞和再细胞化（decellularization and recellularization）肺模型^[41]^。后者指去除肺内所有细胞成分，仅留下细胞外基质的全肺支架作为平台再结合三维培养研究肺干/祖细胞的再细胞化^[42]^。近年，Kajstura等^[30]^首次成功将人肺上皮细胞移植入经低温冷冻的小鼠肺内。Hajj等^[43]^将分离出的囊性纤维化（cystic fibrosis, CF）患者和非CF患者的气道上皮细胞，分别接种至去除上皮的气管移植物中，再种植到裸鼠皮下，发现相比于非CF患者，CF患者的气道上皮细胞存在分化延迟、增殖活跃及基底细胞增生的现象。

### 2.5 慢性标记细胞法

慢性标记细胞法的理论是干细胞在自我更新分裂过程中不对称性分离染色体，引起老的（“永生”）DNA双链保留于子代干细胞而新合成的DNA双链被隔离到正在分化的细胞，从而干细胞通过不对称性染色体分离或因为分裂缓慢而能够保留溴脱氧尿苷（bromodeoxyuridine, BrdU）或[^3^H]胸腺嘧啶等DNA标记物^[44]^。Borthwick等^[9]^用BrdU标记小鼠肺损伤模型，发现标记物主要位于气管和近端支气管的SMGs导管处，推断该处可能存在具有干细胞特征的细胞类型。然而，将此方法用于鉴定干细胞在近年出现了争议。Kiel等^[44]^对新生小鼠、经环磷酰胺和粒细胞集落刺激因子处理的小鼠和成年小鼠给予BrdU 4天-10天，然后停用70天。通过特征性的表面标记高度分离、纯化造血干细胞，发现仅不足6%的造血干细胞保留BrdU，而在BrdU标记的骨髓细胞中造血干细胞少于0.5%，这说明将BrdU作为造血干细胞标记的特异性和敏感性均很低。因此，将BrdU应用于标记气管干细胞尚须进一步验证。

### 2.6 单细胞转录组学分析

单细胞转录组学分析是近年逐渐发展起来的技术，旨在研究不同的时间点单个细胞的增殖、分化、功能及细胞与细胞间相互作用等。此方法不需事先对细胞进行提纯或对潜在的细胞有一定的了解就可直接检测不同的细胞类型和细胞谱系，能用于在分子水平勾画特定细胞类型、鉴定祖细胞和其谱系子细胞以及研究谱系特异的调节因子^[45-47]^。Watson等^[6]^采用单细胞基因表达谱分析发现基底细胞具有异质性，主要由BSCs和BLPCs组成。

## 3 肺上皮干/祖细胞与肺癌

肺癌是世界范围内最常见的致死病因之一，主要根据他的表型和在气道中的不同解剖来源而分为两大类：小细胞肺癌（small cell lung cancer, SCLC）和非小细胞肺癌（non-small cell lung cancer, NSCLC），其中NSCLC占所有肺癌患者近85%，主要分为肺鳞癌、肺腺癌和大细胞肺癌。肿瘤干细胞理论认为肺癌干细胞具有自我更新和分化为肿瘤内所有细胞的能力，是肺癌的发生、进展、复发、侵袭转移、耐药等的“根源”^[48]^。Gu的团队^[49]^首次采用经FACS分离出的小鼠肺干/祖细胞而不是肿瘤细胞系或已形成的肿瘤来研究肺癌干细胞。他们发现多能转录因子Oct-4的过表达足以诱导小鼠肺干/祖细胞的转化。这些转化的细胞不仅具有肿瘤发生和耐药潜能，还显著表达一些促血管生成因子，参与肿瘤血管生成。

大量来自基因工程小鼠模型的数据提示各肺癌亚型可能起源于不同的肺上皮干/祖细胞群。比如，具有神经内分泌特征的SCLC很可能起源于神经内分泌小体内的CGRP^+^ PNECs^[12, 50, 51]^。对于肺腺癌，研究报道SPC^+^CCSP^+^BASCs是在肿瘤基因*Kras*活化后最早增殖的细胞群^[16]^。但是，谱系示踪研究结果却表明Kras^+^肺腺癌可能起源于ATII细胞^[52]^。Desai等^[24]^也观察到EGFR/KRAS信号通路选择性刺激ATII细胞增殖，将ATII细胞转化为快速生长的单克隆肿瘤，他们推测人肺腺癌主要来源于ATII细胞。肺鳞癌具鳞状分化的特性，并常表达基底细胞的表型p63、CK5和转录因子Sox2，提示肺鳞癌可能起源于基底细胞^[53]^，但这尚需基因工程小鼠等实验进一步验证。

肺癌干细胞的深入研究有助于开发出特异性针对肺癌干细胞的药物，如拮抗表面分子标记、去除干细胞的龛位、抑制发展干细胞的信号通路等^[54]^，且逐渐从基础走向临床。已发现的用于分离和研究肺癌干细胞的表面分子标记主要有CD133、CD166、CD44、CD90、乙醛脱氢酶（aldehyde dehydrogenase, ALDH）和转录因子Nanog。Alamgeer等^[55]^报道CD133和ALDH1A1的共表达与已行外科切除的早期NSCLC患者的不良预后强相关，这也符合干细胞表面标记的表达水平与复发相关的假设，因他们可间接反映干细胞的自我更新能力。Yeh等^[56]^将抗精神病药甲哌氯丙嗪联合吉非替尼或顺铂对富集的CD133^+^/CD44^+^肺癌干细胞进行处理后，发现这些细胞的肿瘤干细胞相关基因表达下调、Wnt信号通路受到抑制以及化疗敏感性增加。戒酒硫（disulfiram）之前一直被用于治疗酒精中毒，最近作为ALDH抑制剂在肿瘤治疗领域受到关注。戒酒硫已被报道在多种实体瘤中具有抗肿瘤特性，包括肺癌。Nechushtan等^[57]^进行了一项Ⅱ期临床试验评估在顺铂和长春瑞滨化疗基础上加用戒酒硫治疗晚期NSCLC患者的疗效，结果发现试验组患者的生存期延长。有意思的是，在所有试验者中有两例长期存活者，且这两例均位于戒酒硫试验组。一些高度保守的信号通路主要包括Wnt/β-catenin信号通路、Sonic hedgehog信号通路和Notch信号通路，对于维持正常干细胞的功能至关重要，而肿瘤干细胞常表现出上述一条或多条信号通路的持续活化^[58]^。目前已研制出多种针对这些信号通路的单克隆抗体、siRNA或小分子抑制剂，具有抗肿瘤作用或增强肿瘤对现有治疗的敏感性。一项关于DKN-01在复发或难治性NSCLC患者中的抗肿瘤活性的I期临床研究（NCT01457417）已完成。DKN-01是细胞外dickkopf-1（DKK-1）的高亲和力中和单克隆抗体，而DKK-1是经典Wnt/β-catenin信号通路的抑制剂。NCT01457417的临床研究结果^[59]^表明这些NSCLC患者的中位无进展生存期为2.2个月，总生存期为6.6个月，其中一个患者得到疾病的完全控制。SMO是细胞表面G蛋白偶联受体，Sonic hedgehog信号通路通过SMO的激活启动信号级联反应。今年，一些新研发的小分子SMO拮抗剂，比如LDE-225（NCT01579929）、GDC-0449（NCT00887159）及BMS-833923/XL139（NCT 00927875），已经进入治疗广泛期SCLC患者的临床试验。Notch信号通路抑制剂的研究主要集中于γ-分泌酶抑制剂（γ-secretase inhibitors, GSIs）。γ-分泌酶可将Notch的胞内结构域从细胞膜上释放出来，使之进入细胞核内参与Notch靶基因的表达。GSI不仅具有直接的抗肿瘤活性，还能增强肿瘤细胞对现有治疗的敏感性。GSI可以阻止放疗诱导的Notch通路的活化，提示GSI的应用可能阻止Notch诱导的放疗耐受^[60]^。Arasada等^[61]^发现EGFR突变的肺癌细胞系（HCC4006, HCC827）在接受厄洛替尼处理后，通过*EGFR*依赖的Notch3活化富集了ALDH^+^干细胞样细胞，而GSI可逆转这种表型。

## 4 展望

肺上皮干/祖细胞的研究可能将会集中在鉴定特异的细胞表面标记以富集同质性肺上皮干/祖细胞群，以及采用各种体内体外实验或发展新的功能性分析方法全方位验证各上皮干/祖细胞群的自我更新和分化潜能。同时，明确肺癌干细胞、肺上皮干/祖细胞、肺上皮干/祖细胞龛位之间的关系及相关的信号通路也至关重要。肺上皮干/祖细的深入研究不仅对于明确肺癌在内的肺部疾病的发生发展过程有重要意义，而且在再生医学、靶向治疗等领域对于肺癌在内的难治性肺部疾病的治疗提供了一种新的方向。
